# Under-Five Mortality in Buea Health District, Southwest Cameroon: Evidence from a Community-Based Birth Cohort Study of Rate, Causes, and Age-Specific Patterns

**DOI:** 10.1155/2020/9605492

**Published:** 2020-05-02

**Authors:** Ettamba Agborndip, Benjamin Momo Kadia, Domin Sone Majunda Ekaney, Lawrence Tanyi Mbuagbaw, Marie Therese Obama, Julius Atashili

**Affiliations:** ^1^Health Education and Research Organisation (HERO), Buea, Cameroon; ^2^Faculty of Epidemiology and Population Health, London School of Hygiene and Tropical Medicine, London, UK; ^3^Department of Internal Medicine and Paediatrics, Faculty of Health Sciences, University of Buea, Buea, Cameroon; ^4^Faculty of Medicine and Biomedical Sciences, University of Yaounde 1, Yaounde, Cameroon; ^5^Department of Public Health and Hygiene, Faculty of Health Sciences, University of Buea, Buea, Cameroon

## Abstract

**Background:**

Updating the knowledge base on the causes and patterns of under-five mortality (U5M) is crucial for the design of suitable interventions to improve survival of children under five.

**Objectives:**

To assess the rate, causes, and age-specific patterns of U5M in Buea Health District, Cameroon.

**Methods:**

A retrospective cohort study involving 2000 randomly selected households was conducted. Live births registered between September 2004 and September 2009 were recorded. The under-five mortality rate (U5MR) was defined by the number of deaths that occurred on or before 5 years of age per 1000 live births. Causes of death were assigned using the InterVA-4 software.

**Results:**

A total of 2210 live births were recorded. There were 92 deaths, and the U5MR was 42 per 1000 live births. The mean age at death was 11 ± 15.9 months. The most frequent causes of death were neonatal causes (37%), malaria (28%), and pneumonia (15%). Deaths during infancy accounted for 64.1% of U5M, with 43.5% neonatal (86% occurring within the first 24 hours of life) and 20.7% postneonatal. The main causes of death in infancy were birth asphyxia (37.5%), pneumonia (17.5%), complications of prematurity (10%), and malaria (10%). Child deaths accounted for 35.8% of U5M. Malaria, pneumonia, and diarrhoeal illnesses accounted for the majority of child deaths.

**Conclusions:**

Almost half of U5M occurred during the neonatal period. Improvements in intrapartum care and the prevention and effective treatment of neonatal conditions, malaria, and pneumonia could considerably reduce U5M in Buea.

## 1. Background

Worldwide, remarkable progress has been made in child survival. Between 1990 and 2013, less than 5 million deaths were reported, as well as a reduction in under-five mortality (U5M) by approximately 47% [1, 2]. Thereafter, improved survival was maintained as 1 in 26 children died before reaching the age of five in 2017, compared to 1 in 11 in 1990 [3]. Furthermore, the annual rate of reduction in U5M globally increased from 1.9 per cent in 1990–2000 to 4.0 per cent in 2000–2017 [2]. Despite the global progress in reducing child mortality over the past few decades, an estimated 5.4 million children under age five died in 2017, and about half of these deaths occurred in sub-Saharan Africa [3]. In the region, infant deaths account for approximately 65% of all under-five deaths (34% neonatal deaths and 31% postneonatal deaths) [4].

In Cameroon, the government has been implementing various measures to combat child mortality, such as scaling up the distribution of insecticide-treated bed nets, the Expanded Programme of Immunisation (EPI), and fostering the prevention of mother-to-child transmission of HIV [3]. These actions have been associated with a decrease in the under-five mortality rate (U5MR) from about 146 per thousand in 1990 to 95 per thousand in 2013 and 84 per thousand in 2017, i.e., U5MR decreased by 42% between 1990 and 2017 [3]. Notwithstanding the progress, Cameroon has frequently been ranked among the worst performing countries with respect to progress towards reducing under-five mortality [1, 3]. Moreover, the trend has not been homogenous in all regions of the country. Between 1998 and 2004, while the U5MR dropped in 3 regions, there was a rise in U5MR in the East, Littoral, West, South, and Southwest regions, while in the North and Northwest regions, the rate remained fairly constant. In the Southwest region, the U5MR increased from 96 to 144 per 1000 live births between 1998 and 2004 [5].

The U5MR is a vital indicator of child health, but hidden within it are two important variables: the causes of death and distribution of mortality by age. Although the U5MR is relatively well known in Cameroon, the causes and age-specific pattern of mortality are much more uncertain [1]. Producing accurate estimates and characterization of child mortality remains a considerable challenge in Cameroon and other African countries. This has been attributed to the lack of fully functioning vital registration systems that accurately record all births, and deaths, especially in settings with deep cultural issues surrounding child mortality [1, 6]. Cameroon has legislation for compulsory registration of deaths, but this has still not been adequately implemented in the Southwest region of the country. Proper documentation and management of health information pertaining to child mortality is necessary for the effective planning and evaluation of child health care programmes. The current study is aimed at providing data to supplement knowledge base on the rate, causes, and patterns of under-five mortality in a suburban area in the Southwest region of Cameroon in order to adequately inform the design of interventions and allocation of resources geared towards improving survival of under-five children in this setting.

## 2. Methods

### 2.1. Study Design and Setting

The study was a community-based retrospective cohort study. Field work for the study was carried out over a period of one month (8^th^ December 2014-9^th^ January 2015). The study was carried out in the BHD, situated in Fako division of the Southwest region of Cameroon. Buea is the administrative capital of the Southwest region of Cameroon. The town is located at the base of Mount Cameroon and has 85 villages. It has a regional hospital (secondary level) which serves as the region's referral hospital, 7 primary care facilities, and a few private hospitals.

It shares boundaries with Mutengene to the south, Mount Cameroon to the west, and Ekona to the east. The BHD consists of 7 health areas, namely, Bokwango, Bova, Buea Road, Buea Town, Molyko, Muea, and Tole. BHD covers a total surface area of 870 km^2^. It had an estimated population of 81,478 inhabitants (2010), living in about 37,995 households at the time of the survey. Of this population, children less than five years of age made up an estimated 12% (9778).

For the purpose of this study, BHD was arbitrarily divided into “semiurban” (Buea Road, Muea, and Molyko) and “rural” areas (Bokwango, Bova, Tole, and Buea Town) based on the population size per health area.

### 2.2. Study Population and Sampling

The target population was children born alive to a woman living in a household in the BHD within the period of September 2004 and September 2009. The choice of the assessment period was to allow for each child to be observed for the full five-year period needed for determining under-five mortality. All households with live births within the relevant birth cohort and who gave consent to take part in the study were enrolled for the study. Eligible households where parents or primary carers were absent on two visits were excluded.

Administrative clearance was obtained from the Southwest Regional delegation of Health, as well as the Buea District Health Service. The chiefs of the health centres in the various health areas were informed of the study, and permission was sought from the chiefs of the various villages before going into the community. As this research was conducted as part of a thesis, there was a defined period set to initiate the study, but there was a delay in obtaining authorization to enter communities in three of the seven health areas of the district. A multistage sampling method was employed as follows:

*Stage 1*: the remaining four of the seven health areas of the BHD were selected by convenience: 2 were rural (Bokwango and Buea Town) and 2 semiurban (Muea and Buea Road).

*Stage 2*: from each of the selected health areas, 2 quarters were randomly selected.

*Stage 3*: all households in the selected quarters were approached, and all households with live births within the period of September 2004 and September 2009 were included in the study.

The sample size was calculated using the formula for estimating proportions [7]:

(1) $$\begin{array}{c} n=\frac{z^{2}\times p\left(1-p\right)}{d^{2}}, \end{array}$$

where *n* is the minimum required sample size, *z* is the standard normal variate at a level of confidence of 95% (1.96), and *p* is the prestudy estimate of the under-five mortality rate. We used the estimate obtained in Cameroon for the year 2012 (95/1000) [1]. *d* is the margin of error (0.02):

(2) $$\begin{array}{c} \text{Sample size}=\frac{1.96^{2}\times 0.095\left(1-0.095\right)}{0.02^{2}}=825. \end{array}$$

The population of children less than five years of age in the BHD was 9778; therefore, the corrected sample size was calculated as follows:

(3) $$\begin{array}{c} n=\frac{n_{0}}{1+\left(n_{0}/N\right)}, \end{array}$$

where *n* is the corrected sample size, *n*_0_ is the original sample size, and *N* is the total population of children under five years of age:

(4) $$\begin{array}{c} \text{Sample size}=\frac{825}{1+\left(825/9778\right)}=760. \end{array}$$

From this formula, a minimum of 760 live births were required for the study. A minimum of 760 households with a live birth between September 2004 and September 2009 were therefore required for the study. To increase precision, 2000 households were assessed.

### 2.3. Ethical Considerations

Ethical approval was from the Institutional Review Board of the Faculty of Health Sciences, University of Buea, Cameroon, under the registry number 2014/260/UB/FHS/IRB.

### 2.4. Data Collection

The members of the research team included the principal investigator and ten fourth year medical students who had successfully completed a community health practice course. They were trained for 1 week on the study questionnaire before going into the community. A separate interview was carried out for each case of mortality observed in a household. The participants were met in their homes every weekday from 4 to 6pm. The purpose of the study was explained to them, and those who were willing to participate and gave their consent were included in the study.

We used a data collection form to record the number of live births within the study period, in each household, and the number of those children (if any), who died before their fifth birthday. In houses where a child had died, the study questionnaire (interviewer-administered) was administered, in order to ascertain the cause of death.

The first portion of the questionnaire assessed the age at death, gender, birth order, level of education of parents, use of health facilities, and place of death and whether or not death was certified. The second portion of the questionnaire, the section on verbal autopsy, asked precise questions about the complaints, as well as the signs, and symptoms that preceded the child's death.

### 2.5. Definition of Terms and Concepts

*Neonatal mortality rate*: the number of infant deaths between 0 and 28 days, per thousand live births [8].

*Postneonatal mortality*: deaths between the ages of 28 days and 12 months [1].

*Infant mortality rate*: the probability of dying during the first year of life, per thousand live births [1].

*Under-five mortality rate*: the probability of dying between 0 and 5 years, per 1000 live births [1].

*Child mortality rate*: the probability of dying between 1 and 4 years, per thousand live births [1].

### 2.6. Data Management and Statistical Analysis

At the end of each day, the total numbers of births within the study period, as well as the total number of deaths registered for that day, were entered into Microsoft Excel 2007. All questionnaires were assembled by the primary investigator and checked visually for completeness, obvious errors, and inconsistencies. Data generated from the study questionnaire was entered into Microsoft Excel 2007 and transferred to the software package EPI INFO for Windows, Version 6.40 for analysis.

To attain objective 1, we calculated U5MR using the following formula:

(5) $$\begin{array}{c} \frac{\text{Total number of deaths occurring on or before }5^{\text{th}}\text{ birthday between }2004\text{ and }2009}{\text{Total number of live births between }2004\text{ and }2009\;}\times 1000. \end{array}$$

The mortality rates for different maternal and child characteristics were compared using a chi-squared test.

Regarding objective 2, we assigned the causes of mortality by using the InterVA-4 software, which is a Bayesian probabilistic model for interpreting verbal autopsy (VA) data. The frequencies of the various causes of death were described. The InterVA model was recently developed as a computerised model for interpreting verbal autopsy. Given a set of indicators, it works by calculating the probability of certain causes of death, based on the signs and symptoms obtained from the verbal autopsy interview. It has different levels of sensitivity and specificity for different populations, depending on the causes of morbidity and death in said populations.

Concerning objective 3, we analysed the data using EPI INFO for Windows, Version 6.40, and the frequencies and percentages of deaths which occurred among the various age groups were calculated.

## 3. Results

A total of 2000 households were visited. In these households, a total of 2210 live births were recorded between September 2004 and September 2009. Among the live births, 92 children died before the age of 5 years. Nonrespondents were not recorded for the households, and the birth cohort was complete.

### 3.1. Characteristics of Under-Five Deaths

#### 3.1.1. Age

The mean age at death was 11 ± 15.9 months, and the median age at death was 5 months.

#### 3.1.2. Sex

Fifty-seven (61.9%) of the 92 under-five deaths were males.

#### 3.1.3. Maternal Age

Forty deaths (43.47%) occurred among children whose mothers were less than 20 years old, 41 deaths (44.57%) occurred among children whose mothers were aged between 20 and 35 years old, and 11 (11.96%) deaths occurred among children whose mothers were more than 35 years old, *p* < 0.005.

#### 3.1.4. Maternal Education

Of the 92 deaths, 35 (38%) occurred among children whose mothers had attained primary education, and 36 (39%) among those whose mothers had secondary education.

#### 3.1.5. Health-Seeking Behaviour

All of the children were born in health facilities, and formal medical care was sought for 84 (91%) during the illness that led to death. Two (2.17%) of children died without access to any form of medical care. The rest (7%) were treated at home with medication from drug stores. Formal medical care was sought for all neonatal deaths.

#### 3.1.6. Certification of Deaths

Of the 92 deaths, death certificates were only obtained for 5 (5.43%) children.

#### 3.1.7. Residence

Majority of deaths were among children living in the Muea health area (40.2%), while the least number of deaths was recorded among those living in Buea Road health area (14%).

### 3.2. Under-Five Mortality Rate

We recorded a total of 2210 live births and 92 deaths. The under-five mortality rate was found to be 42 per 1000 live births (95% CI (33, 50)). Under-five mortality in this study has been divided into two main age groups: infant mortality (neonatal and postneonatal) and child mortality.  Table 1 stratifies the under-five mortality rate by age groups.

Table 2 summarises the main background characteristics of children who died before the age of five. The under-five mortality rate was higher in the rural health areas compared to the semiurban health areas. The under-five mortality rate was highest among male children, children born to mothers aged less than 20 years, and children whose mothers had no education.

### 3.3. Causes of Under-Five Mortality

Verbal autopsy reports were obtained for 70 of the 92 deaths. For the other 22 cases, the informants could not provide enough data or were unwilling to talk about the circumstances of death. The cause of death for 7 cases could not be identified by the Inter-VA4 software. As concerns the remaining 63, the causes of death were neonatal causes (birth asphyxia, prematurity, and neonatal sepsis) (37%), malaria (28%), pneumonia (15%), diarrhoea (7%), meningitis (7%), and others like accidents (6%).

### 3.4. Age-Specific Patterns of Under-Five Mortality

#### 3.4.1. Infant Mortality

Deaths during infancy accounted for 64.1% of all under-five deaths. The main causes of infant mortality were birth asphyxia (37.5%), pneumonia (17.5%), prematurity (10%), and malaria (10%).

#### 3.4.2. Neonatal Mortality

Of all the under-five deaths registered in our study, neonatal deaths accounted for 43.5%. Of the 40 neonatal deaths, 32 (80%) occurred within the early neonatal period, and 86% of early neonatal deaths occurred within the first 24 hours of life. Birth asphyxia was the principal cause of neonatal deaths. The main causes of neonatal mortality were birth asphyxia (66%), complications of prematurity (14%), neonatal sepsis (10%), meningitis (7%), and pneumonia (3%).

#### 3.4.3. Postneonatal Mortality

Nineteen (20.7%) of under-five deaths occurred in the postneonatal period. The main causes of death in this age group were pneumonia (31%), malaria (31%), meningitis (23%), and diarrhoea (15%).

#### 3.4.4. Child Mortality

Thirty-three (35.8%) of under-five deaths occurred between 1 and 5 years. The causes of death in this age group were malaria (54%), pneumonia (15%), diarrhoea (12%), and meningitis (8%).

## 4. Discussion

This study was carried out to assess the rate, causes, and age-specific patterns of under-five mortality in Buea Health District, located in the Southwest region of Cameroon. We recorded ninety-two deaths, and the under-five mortality rate was 42 per 1000 live births. The most frequent causes of death were neonatal causes, malaria, and pneumonia. Infant mortality accounted for most of the deaths, with almost half being in the neonatal period. The main causes of death in infancy were birth asphyxia, pneumonia, complications of prematurity, and malaria. Child deaths accounted for a little more than a third of under-five deaths, with malaria, pneumonia, and diarrhoeal illnesses accounting for the majority of deaths in this age group.

This study is among the few reports that address the crucial lack of population-based data on mortality in children below the age of five in Cameroon. The study contributes to filling the knowledge gap in under-five mortality by providing evidence from a large-scale community-based survey. The large number of participating households and the use of multistage random sampling to enrol these households ensured that there was good representation of the target population. Additionally, this study provides details on mortality data among all and specific subgroups of children in the age range 0-59 months in a Cameroonian setting. The large proportions of neonatal deaths (mainly observed within the first 24 hours of life) and death from specific causes such as pneumonia and malaria reveal avenues to avert important preventable causes of mortality among children under the age of five in the study setting.

More than a third of under-five mortalities in our study occurred within the first month of life, and the principal causes were neonatal causes and malaria. U5M was frequent in males and children of young mothers and when maternal education was poor. The U5MR in our study was much lower than the U5MR in a previous major report which showed that by 2004, U5M in the Southwest region of Cameroon was 144/100 [5]. Certain factors could explain this large difference. The U5M estimated by the preliminary report was decennial, i.e., it captured U5M that occurred from 1995 to 2004 in the whole region while our study focused on the birth cohort of September 2004-September 2009 in BHD. Furthermore, the previous report was a multiregional assessment of the U5MR and therefore included mortality data from all districts in the Southwest region, including those that experience enormous shortfalls in healthcare resources relative to BHD. It is worth noting that BHD has the Buea Regional Hospital which is staffed with specialists, including paediatricians and emergency care physicians, and this hospital is the largest referral centre in the Southwest region. Furthermore, compared to other districts, BHD has better water supply, and its good road network facilitates access to the available health facilities.

The U5MR in our study was lower than what was observed in previous large-scale studies in East and South Africa. One of these assessed the two-year mortality rate for a community-based cohort of 2500 infants enrolled from birth up to 8 weeks of age during the periods November 2008-November 2009 in Uganda [8]. The study reported an U5MR of 60/1000. The other study, which involved data from multiple databases in Kenya, reported a decline in U5MR from 113 to 79 during the period 1993-2008 [9]. These higher U5MR could be attributed to the fact that these previous studies involved economically disadvantaged settings in urban slums and purely rural areas. The third study which was conducted in South Africa was an analysis of a population-based cohort in rural KwaZulu-Natal and found that for the period 2000-2014, the U5MR was 58/1000 [10]. The higher U5MR in this cohort could be explained by the hyperendemicity of HIV (and high rates of HIV-related mortality) in this setting. Nonetheless, the above study reported an overall decline in the trends of U5MR between 2000 and 2014, and this was attributed to the marked improvements in immunisation rates, social grants, and nutrition in South Africa, and importantly, the roll-out of antiretroviral therapy significantly reduced the burden of HIV which was a leading cause of death in KwaZulu-Natal prior [10].

The observed U5MR among children born to mothers aged less than 20 years is consistent with the aforementioned report from Uganda [8] and a large-scale study that sought to identify causes of death in Iraq during the period 1994-1999 [11]. But in a large secondary analysis of 2011 data from upper eastern Ghana [12] and according to the 2007-2010 Demographic Health Survey data for Lesotho and Liberia [13], U5MR was the highest for children born to mothers aged >35 years. Although both teenage motherhood and advanced age have been generally linked with infant mortality, the above differences in the contributions of maternal age to U5MR suggest that demographic determinants of U5M may tend to be region-specific. The observation of more deaths in the rural than in the semiurban settings may be linked with factors such as lack of education and lower economic power in the former settings and has been reported in other African countries [8, 9]. However, performing subgroup analyses of risk factors for U5M in order to assess possible reasons for the significantly higher U5MR in rural compared to semiurban settings was not within the scope of our study. Although U5MR tends to be higher in rural compared to urban settings, a large-scale study of U5MR during the period 1993-2008 in Kenya showed that differences in U5MR between urban and rural settings narrowed with time [9]. This unusual observation was attributed to the development of urban slums where the deplorable living conditions are comparable to those of rural areas. The significantly higher U5MR in children whose mothers had no formal education has been extensively reported in diverse African settings [10, 13–17], and our findings align with these.

The main causes of under-five mortality in our report are consistent with the causes of under-five mortality that have been generally reported in Cameroon and results from previous studies in Ghana [18], Mozambique [19], and Ethiopia [2]. However, our findings differ from reports in Nigeria [20], China [21], and even on the global scale [22]. The difference observed between our study and these latter reports could be due to variations in study settings, heterogeneity in disease epidemiology, and the more advanced levels of health infrastructure in these reports. As in our study, a previous study by Chelo et al. in Cameroon found that death due to asphyxia accounted for the majority of neonatal deaths [23]. However, this previous study found that the neonatal mortality rate was just a third of the neonatal mortality rate observed in our study, and this is possibly because Chelo et al. analysed data on newborns in a level one maternity and focused on early neonatal deaths for the period August 2007-January 2008 [23]. Out of Cameroon, analyses of data from a large population-based cohort in South Africa for the 2000-2014 period also found that deaths among neonates contributed most to U5M and the neonatal mortality rate (21/1000) was comparable to that in our study [10]. Contrary to our study, previous studies in Nigeria [20] and South Africa [24] reported that mortality in children of older age groups contributed most to U5M. In the Cameroonian setting, neonatal deaths being the main cause of U5M could be the result of suboptimal emergency obstetric and neonatal care services [25], and similar conditions were likely experienced in the Ugandan setting, while the population-based South African study was conducted in an HIV hyperendemic rural setting [10]. The contrary reports provided by the hospital-based Nigerian and South African studies could be attributed to the study setting since these studies were conducted in tertiary care centres with adequate capacities for emergency obstetric care and neonatal resuscitation. Based on the aforementioned studies from diverse settings, it can be argued that in a low-income setting, data on under-five death from hospital-based studies, especially the highly resourced facilities, may not be adequately representative of the actual mortality data in that low-income setting. In our study, malaria was a major cause of mortality in both infants and children. Malaria is endemic in Cameroon and in BHD; evidence of low coverage and underutilisation of insecticide-treated bed nets have been reported [26]. Similar barriers may have reduced the effectiveness of other vector control measures (such as in-door residual spraying) and other important interventions geared towards malaria prevention such as chemoprophylaxis in infants and pregnant women in this setting.

Our study has some limitations. The determination of causes of death through the verbal autopsy method was based on reports from relatives of the deceased children. Verbal autopsy is useful in elucidating the probable cause of death, but the tool is less accurate for diseases with nonspecific symptoms like malaria. Again, while noting that the InterVA software was useful in tracking mortality patterns in our study, the software may not always indicate the cause of death as we observed in our analysis. This factor must have led to measurement bias and reduced the internal validity of our findings. Emotional states of respondents and the sensitive nature of the information collected may have contributed to respondent and social desirability biases. Furthermore, meeting with respondents long after the death of a child could have been associated with some recall bias, but in order to improve precision, we collected information in days for neonatal deaths, in months for infants, and in years for the rest, and importantly, as explained in the methods, we expanded our estimated sample size from a minimum of 760 to 2000 households which helped limit the imprecision induced by informants who could not provide enough data or were unwilling to talk about the circumstances of death. Significant delays in obtaining authorization to enter three health areas contributed to sampling bias. In order to have a considerable sampling frame despite this limitation, we conveniently enrolled communities in the remaining four health areas and then proceeded to do a multistage sampling to reduce sampling bias. Alterations in the population composition, for instance, due to factors such as migration, could not be assessed in this study. Nonetheless, quarters in each health area of Buea tend to be in very close proximity and have very similar socioeconomic, environmental, and overall living conditions. Under these circumstances, the possibilities of alterations in the population structure and clustering due to the random selection of two quarters per health area were limited, and the birth cohort was likely representative of the population of BHD.

## 5. Conclusion

The aim of our study was to assess the under-five mortality rate in the BHD as well as the major causes and age-specific patterns of mortality. We found that the U5MR in the BHD was 42 per 1000 live births. The U5MR was the highest among male children, children born to mothers aged less than 20 years, children born to mothers who had no form of formal education, and children born in rural health areas. The main causes of U5M in the BHD were neonatal causes, malaria, and pneumonia. Majority of under-five deaths occurred in the neonatal period, with most deaths occurring within the first 24 hours. And given that the leading cause of neonatal death was asphyxia, it is likely that effective neonatal care services and maternal care (notably, intrapartum care) could be primordial in averting mortality in BHD. Prevention of malaria, pneumonia, and diarrhoea, as well as early and effective treatment of these illnesses, could be key interventions to reduce under-five mortality in this setting.

## Figures and Tables

**Table 1:** Mortality rate stratified by age groups.

Age group	Number of deaths (percentage)	Mortality rate (/1000 live births) (95% CI)
Infant	59 (64.1%)	27 (20-34)
Neonatal	40 (43.5%)	18 (13-24)
Postneonatal	19 (20.6%)	09 (5-13)
Child	33 (35.9%)	15 (10-20)
Under-five	92 (100%)	42 (33-50)

**Table 2:** Under-five deaths categorised by maternal and child characteristics.

Characteristics	Live births	Under-five deaths (percentage)	U5MR (per 1000 live births)	*p* value
Health area				
Bokwango	303	25 (27.2%)	82.5	<0.0001
Buea Town	243	17 (18.5%)	70	
Buea Road	653	13 (14.1%)	19.9	
Muea	1011	37 (40.2%)	36.6	
Type of health area				
Rural	546	42 (45.7%)	77	<0.0001
Semiurban	1664	50 (54.3%)	30	
Gender				
Male	1193	57 (61.9%)	48	0.17
Female	1017	35 (38.1%)	35	
Maternal age (years)				
<20	515	40 (43.4%)	78	<0.0001
20-35	1112	41 (44.6%)	37	
>35	583	11 (12.0%)	19	
Maternal education				
None	199	18 (19.6%)	90	<0.0001
Primary	464	35 (38.1%)	71	
Secondary	884	36 (39.0%)	41	
Tertiary	663	03 (03.3%)	5	

## Data Availability

All data generated or analysed in this study are available from the corresponding author upon reasonable request

## Abbreviations

BHD: Buea Health District
U5M: Under-five mortality
U5MR: Under-five mortality rate.
